# To Ski or Not to Ski on Replaced Hips

**Published:** 1990-12

**Authors:** Michael Wilson


### From our correspondents

### TO SKI OR NOT TO SKI—THAT IS THE QUESTION FOR SKIERS WITH REPLACED HIPS

### A report on two friends

Since the outbreak of peace and prosperity in Europe forty years ago skiing has become one of the most popular pastimes, and now many of its ageing devotees have had their hips replaced, they are still sound in wind, are they also sound in limb? They feel physically restored, the snows and the slopes beckon, should they accept the challenge or should 'wiser councils prevail'?

My interest in this question arises because the dilemma has faced two of my friends and skiing companions of many years. The first Maurice Willoughby, started skiing before the war at the age of 16. A regular army officer, he was at one time an instructor in the School of Mountain Warfare at The Cedars in the Lebanon, where he taught me to ski in 1946. He later represented Great Britain in the Winter Pentathlon in the first post-war Olympic Games at St Moritz, where, incidentally, the British was the only pentathlon team in which every member completed all the events. He has skied virtually every year since, is registered as a skiing instructor in both Britain and Austria, and for many years, as skiing corresponding of the *Daily Mail* has spent several months in the Alps each year reporting on the scene. At the age of 70 he was the star of BBC Television series in which he showed that you could still take up new sports at that age—some readers may remember the 'Willoughby' programme. In 1984, aged 71, he began to have some pain in the R hip on walking and an X-ray showed Osteo-arthritis of both hips, more severe on the Rt. Gradually this became incapacitating and late in 1987 he had a R hip replacement. In 1988 he could not consider skiing, in 1989 he was frustrated by lack of snow in the Alps. In 1990 he decided to try again after seeking the advice of his consultant orthopaedic surgeon, who wrote to him as follows.

"First of all I have not seen a patient who has come to harm after a hip replacement. However, clearly anybody with a hip replacement must be more at risk than anybody with a normal hip. Not only that, but a fracture sustained in proximity to an artificial joint is always more difficult to deal with. For these reasons alone, I therefore do not encourage my patients to ski. Having said that, many patients do ski and those who are very proficient seem to come to no harm. By the very nature of things, people keen to ski in middle and old age are almost certainly experts and, therefore, the risks from accidental injury are correspondingly much less than in the younger, less experienced people albeit with better hips. Obviously there is a somewhat increased risk in skiing with an artificial hip, but probably the risk is not very great."

Pondering this advice Maurice decided to ski again in 1990. His knowledge of skiing conditions in the Alps must be almost unrivalled, because, as skiing correspondent of the *Daily Mail* he has skied that area year after year, reporting on the resorts large and small, famous and little known.

A number of factors contribute to skiing accidents and many of these are avoidable. Among them are-

1) Collisions. Crowded slopes and bottle necks in the pistes bring a lot of people travelling fast in close proximity. Collisions become unavoidable, two people falling may then produce a pile up and anything may happen, (though fortunately it seldom does). Therefore avoid overcrowded resorts, school holidays and narrow forest paths where there is no room for manoevre.
2) Visibility. Many falls result from poor visibility due to cloud or falling snow. These conditions prevail in December and January.
3) Icy conditions. As the sun gets hotter in late March and April the surface snow melts at midday and then freezes later in the afternoon leaving an icy surface. Skiing can then be difficult before eleven o'clock and after four.
4) Steep slopes are obviously more difficult than gentle ones. In some resorts the mountains are steeper, the gradients more difficult and the pistes narrower than others.

Choice of resort and of month is therefore important in making for an accident free holiday. Bearing in mind these considerations my friend chose Austria in early March. He chose Austria because on the whole altitudes are optimal and difficult slopes are rare, unlike many Swiss resorts. He chose an area in which a number of small villages have access to a central massif, where they are all interconnected by a large number of lifts and the skiers are spread out over a large area. He chose early March because one can expect sun and good visibility and the snow should not have started melting. He also prefers the small family run hotel because these are friendly and very good value for money. The final result of all these computations was Ellmau in the Tirol on the fringe of the Grossraum, the biggest interconnected ski system in Austria, and the Haus Bavaria, a charming newly built small family-run hotel. The choice proved very satisfactory though sunshine melted the lower slopes in the second week. He also decided to have as companions two doctors, a physician and a surgeon whose professional advice he hoped not to need.

The other friend is perhaps at the opposite end of the scale in skiing experience. Now 66 he did not start skiing until the age of 54. As Colonel Willoughby started me off on the ski slopes, so did my rapturous accounts of skiing holidays persuade Dr John Inskip to 'have a go' well into his 6th decade. He took to it immediately becoming rapidly very proficient and skied every year until 1988 when pain and limitation of movement in both hips persuaded him to have both his hips replaced. He missed skiing in 1989 but in 1990 decided to throw caution to the winds and ski again. He chose to go to Valmorel but was diverted because of lack of snow to Les Deux Alpes in France.

How did my friends fare? I was with Maurice Willoughby, he skied carefully and very stylishly for the first week, but the snow was rather heavy and while the replaced hip gave no trouble the unoperated side began to be painful and limited his skiing during the second week. John Inskip had a splendid holiday and thought he had never skied better, with absolutely no trouble from his hips. He prepared himself for the holiday only by playing golf regularly, which he says he now plays better than he has done for 10 years.

While in Ellmau I decided to pay a visit to Dr George Leitner, the local doctor to whom in the first instance most of the skiing injuries were brought. He was quite forthright in his views. He said "Old men should not ski downhill, they should become langlaufers". He said that treatment of patients who have fractured their femurs at the site of insertion of the prosthesis can be very difficult and can end with the leg being shortened. He was very positive in his opionion and Maurice Willoughby who was with me and having pain in his other hip was quite discouraged.

A couple of months later I travelled with the Moynihan Surgical Club to Norway and at Bergen heard Dr Lars B. Engesaeter speak on the "National Registry for Hip prostheses—the first 12,000 patients". These patients had all been followed up and the results classified. They had used Charnley's prosthesis with mainly excellent results. 145 patients had needed revision. 99% of the prosthses were still in position. I had an opportunity of asking Dr Engesaeter what advice he gave to patients who wished to continue skiing. Without hesitation he replied that he encouraged them to do so. Skiing in Norway is mainly langlaufing but there is plenty of downhill skiing as well. I did not ask him whether he advised them to give the Holmenkollen a miss.

As a postcript to this little report both Colonel Willoughby and Dr Inskip intend to ski again in 1991 and I hope to get them together at Ellmau.

MICHAEL WILSON

